# Probiotic, Prebiotic, and Synbiotic Supplementation for the Prevention and Treatment of Acute Otitis Media: A Systematic Review and Meta-Analysis

**DOI:** 10.3390/children12050591

**Published:** 2025-04-30

**Authors:** Freiser Eceomo Cruz Mosquera, Mayerli de la Rosa Caldas, Anisbed Naranjo Rojas, Claudia Lorena Perlaza, Yamil Liscano

**Affiliations:** Grupo de Investigación en Salud Integral (GISI), Department of Health Sciences Faculty, Universidad Santiago de Cali, Cali 760035, Colombia; mayerlide00@usc.edu.co (M.d.l.R.C.); anisbed.naranjo00@usc.edu.co (A.N.R.); lorena.perlaza00@usc.edu.co (C.L.P.); yamil.liscano00@usc.edu.co (Y.L.)

**Keywords:** probiotics, prebiotics, dysbiosis, microbiota, systematic review, acute otitis media

## Abstract

Background and Aim: Probiotics, prebiotics, and synbiotics have been documented to modulate the microbiota, enhance immunity, and reduce antibiotic resistance, making them a promising alternative in the management of acute otitis media (AOM). Accordingly, the aim of this study was to determine their effectiveness in the prevention and treatment of AOM in patients. Methods: A systematic review and meta-analysis of randomized controlled trials published between 2000 and 2024 was conducted using Science Direct, PubMed, LILACS, SCOPUS, Web of Science, and Cochrane Clinical Trials, following PRISMA guidelines. The methodological quality was evaluated using the Jadad scale, and the meta-analysis was performed with RevMan 5.4^®^ and Jamovi 2.3.28^®^. Results: A total of 16 trials with 4034 patients were included. The meta-analysis showed that the intervention did not affect the time to AOM presentation (MD: −7.98; 95% CI: −19.74 to 3.78; *p* = 0.18), the recurrence of the disease (RR: 0.99; 95% CI: 0.74–1.33; *p* = 0.96), or the requirement for antibiotics (RR: 1.31; 95% CI: 0.92 to 1.84; *p* = 0.13); however, it was associated with a reduced probability of developing AOM (RR: 0.80; 95% CI: 0.66 to 0.96; *p* = 0.02). Subgroup analysis suggests that the effect of probiotic supplementation on AOM incidence is influenced by treatment duration, patient age, and the number of probiotic strains in the product. Conclusions: Supplementation with probiotics, prebiotics, or synbiotics is associated with a significant reduction in the incidence of AOM in children, although no significant impact was observed on other key clinical parameters. These interventions may be considered as a complementary strategy to conventional treatments; however, further high-quality, standardized trials are needed to confirm these findings and to define optimal protocols.

## 1. Introduction

Acute otitis media (AOM) is one of the most common infections in children and represents a significant cause of illness in this age group worldwide [1,2,3]. It is characterized by inflammation of the middle ear space and the presence of fluid, which can lead to fluctuating hearing loss [4,5]. AOM typically follows a viral infection of the upper respiratory tract, specifically affecting the nasopharynx and Eustachian tube. The inflammation caused by the virus facilitates bacterial colonization and subsequent infection of the middle ear [6]. Colonization of the middle ear and nasopharynx by bacteria such as *Streptococcus pneumoniae*, *Haemophilus influenzae*, and *Moraxella catarrhalis* is the main risk factor for developing AOM [7,8]. Untimely treatment of AOM can lead to serious complications with varying frequencies, including mastoiditis, hearing loss, meningitis, brain abscesses, sigmoid sinus thrombosis, epidural or intracerebral abscesses, petrous apicitis, and otitic hydrocephalus, carrying a risk of death in extreme cases [9,10].

Acute otitis media is one of the most common reasons children under five years old (up to 75%) seek medical consultation and receive antibiotics, with more than 10 million prescriptions issued annually in the United States [11,12]. Globally, the annual incidence of AOM is estimated at 10.85% per 100 people. This percentage varies significantly by region, being lowest in Central Europe (3.64%) and highest in sub-Saharan Africa (43.37%) [13]. AOM accounts for approximately 30 million medical visits per year and generates additional healthcare costs exceeding $2.5 billion, highlighting the importance of optimizing its management [14].

The treatment for acute otitis media depends on its type and severity, traditionally including observation, antibiotics, and even surgery when complications arise [15]. Some studies suggest AOM is the primary reason antibiotics are prescribed to children in the U.S., accounting for more than 10 million prescriptions annually and representing 24% of all pediatric antibiotic prescriptions [16,17,18,19,20]. A systematic review involving over a thousand children at risk for AOM found that long-term antibiotic treatment might reduce episodes of otitis media [21]. However, the excessive use of broad-spectrum antibiotics significantly contributes to growing antibiotic resistance, dysbiosis, and, in some cases, can lead to more severe reactions without ensuring a reduced recurrence of infection [22,23,24].

In response to these limitations, the role of alternative and complementary strategies has been explored to effectively prevent or treat AOM while minimizing adverse effects associated with traditional management. Among these alternatives, probiotics, prebiotics, and synbiotics have emerged as promising interventions [25,26]. Probiotics are live microorganisms that, when administered in adequate amounts, confer health benefits to the host. These microorganisms, including strains of Lactobacillus, Bifidobacterium, and Streptococcus, may stabilize the microbiota, modulate immune responses, and compete with pathogens in the upper respiratory tract [27]. In the context of AOM, these interventions could play a critical role in its prevention and treatment, as indicated by some studies [28,29]. Nevertheless, it must be acknowledged that evidence regarding the effectiveness of probiotics, prebiotics, and synbiotics in AOM remains controversial due to conflicting results among various studies, some of which are inconclusive.

Given the above considerations, this systematic review and meta-analysis aims to determine the effectiveness of probiotics, prebiotics, and synbiotics for the prevention and treatment of patients with AOM or those at risk of developing it.

## 2. Materials and Methods

### 2.1. Study Protocol

A systematic review with meta-analysis was performed based on the recommendations of the Cochrane Collaboration Handbook [30] and the guidelines of the Preferred Reporting Items for Systematic Reviews and Meta-Analyses (PRISMA) [31,32]. The protocol was previously registered in the International Prospective Register of Systematic Reviews (PROSPERO, https://www.crd.york.ac.uk/prospero/, accessed on 19 December 2024) under the code CRD42024629808. The research was designed considering the population, intervention, comparison, and outcomes (PICO strategy).

### 2.2. Research Question

In patients with acute otitis media or at risk of AOM (P), is the administration of probiotics, prebiotics, or synbiotics (I), compared to placebo or standard care (C), effective in preventing the development of the disease, delaying its onset, reducing recurrence, shortening the duration of the clinical episode, decreasing the requirement for antibiotics, reducing hospitalizations, lowering the incidence of upper respiratory tract infections, and minimizing adverse events (O)?

### 2.3. Eligibility Criteria

#### 2.3.1. Inclusion Criteria

The inclusion criteria considered in the review were as follows:

- Randomized and controlled clinical trials, regardless of follow-up duration and specific study design (crossover, parallel, among others).
- Trials presenting multiple study groups will be included when the arm assigned to treatment with probiotics, prebiotics, or synbiotics can be isolated.
- Research published between January 2000 and July 2024.
- Studies published in English, Spanish, and Portuguese.
- Studies in populations under 18 years old, with acute otitis media or at risk of developing the disease, in which the effectiveness of prebiotics, probiotics, or synbiotics—using single or combined strains, regardless of presentation (capsule, food, fermented, powder), duration of administration, and treatment regimen—is evaluated.
- Studies that consider at least one of the following outcomes: development of acute otitis media, time until its onset, recurrence of the disease (3 episodes in 6 months or 4 episodes in 12 months), duration of the clinical episode, requirement for antibiotics, hospitalizations, incidence of upper respiratory tract infections, and adverse events.

#### 2.3.2. Exclusion Criteria

The exclusion criteria considered in the review were as follows:

- Articles in preprint format.
- Studies published as conference abstracts.
- Articles in the form of letters to the editor with insufficient information.
- Studies not available in an accessible format.
- Studies employing the same cohort of patients used in previous similar investigations.
- Studies that include patients with acquired or congenital immunodeficiency, craniofacial abnormalities, or chronic diseases.
- Studies that do not specify the strain present in the product supplied to the patients.
- Studies using an animal model of AOM.
- Studies in which it is difficult to evaluate the specific contribution of prebiotics, probiotics, or synbiotics to the outcome due to the simultaneous application of other interventions.

### 2.4. Data Sources and Search Strategy

The search was conducted in the following databases: Science Direct, PubMed, LILACS, SCOPUS, Web of Science, and Cochrane Clinical Trials. Filters for Spanish, Portuguese, and English were applied, along with the previously established date when necessary. The search strategy was designed and implemented from December 2024 to February 2025 by three independent researchers (MRC, CLP, ANR), using the following strategy: (“probiotics” OR “gut microbiota” OR “microbiome” OR “symbiotics” OR prebiotics) AND (“acute otitis media” OR “AOM” OR “middle ear infection” OR “ear infection” OR “upper respiratory tract infection” OR “URTI”) AND (prevention OR treatment OR “clinical outcome” OR “efficacy” OR “therapeutic effect” OR “management”) AND (“randomized controlled trial” OR “clinical trial” OR “RCT” OR “controlled trial”).

The strategy was adapted for each database when necessary. Additionally, the bibliographic references of relevant articles were reviewed and a manual web search was conducted to identify studies not found in the initial search. When additional information about a study was needed, data were consulted on ClinicalTrials.gov (https://clinicaltrials.gov/, accessed between 10 January and 15 February 2025) or in the repository indicated in the article as the deposit for the clinical trial protocol. Data storage was performed using Rayyan—Intelligent Systematic Review (https://www.rayyan.ai/, accessed between 10 January and 30 February 2025).

### 2.5. Selection and Data Extraction

The selection of potentially eligible studies was carried out by two independent researchers (YLM, FCM) by evaluating the title, abstract, and subsequently the full text of the article. When the full text could not be accessed, the corresponding author was contacted. Studies with discrepancies were discussed, and the decision to include them in the review was made by consensus. If an agreement could not be initially reached, a third reviewer intervened. To measure the degree of agreement among the authors and to evaluate consistency in study selection, Cohen’s Kappa index was used. Three reviewers (MRC, CLP, ANR) extracted the information from the selected studies, considering the main study data (first author, year of publication, country); characteristics of the participants (number of subjects assigned to each group, age expressed, percentage of male participants); characteristics of the intervention (probiotic, prebiotic, or synbiotic used, presentation, dosage, treatment duration); and outcomes evaluated. Subsequently, other reviewers (FCM, YLM) verified the integrity and accuracy of the recorded information.

### 2.6. Risk of Bias Assessment

The risk of bias in the clinical trials was independently assessed by reviewers FCM and YLM, using a standard tool that covers the key aspects of this type of study [33]. The information collected during the assessment was recorded in Review Manager version 5.4^®^ (RevMan). The evaluation criteria included: (a) random sequence generation, (b) allocation concealment, (c) blinding of participants and personnel, (d) masking of outcome assessment, (e) incomplete outcome data, and (f) selective reporting. For each criterion, the clinical trials were determined to have a low, unclear, or high risk of bias based on adherence to the predefined guidelines. Discrepancies in the risk of bias assessment were resolved through discussions among the reviewers until consensus was reached.

### 2.7. Assessment of the Quality of Evidence

The quality of the studies included in the meta-analysis was assessed using the Jadad scale, which assigns a total score between 0 and 5 (with 5 being the highest score, indicating better quality) based on several criteria: (a) the study is randomized, (b) the intervention is double-blind, (c) dropouts are specified and considered, (d) the randomization procedure is properly conducted using an appropriate method, and (e) the inclusion and exclusion criteria are clearly described. For each criterion, a score of “0” was given if it was not met or if the description was insufficient, and “1” if the criterion was adequately met. A total score between 0 and 2 indicated that the clinical trial was of low quality, while a score of 3 or higher was considered indicative of adequate quality. Although studies were not excluded based on this evaluation, the quality scores were taken into account when reporting the results.

### 2.8. Statistical Analysis

For the meta-analysis, the RevMan 5.4^®^ software was used. The effect size along with the 95% confidence interval was calculated. The meta-analysis was carried out when there were at least two studies that evaluated any of the outcomes considered in the review. The outcomes included in the meta-analysis were as follows: development of AOM, time until disease onset, recurrence of AOM, and antibiotic requirement for the treatment of AOM. For quantitative outcomes, the mean and standard deviation from the primary study were extracted. In cases where results were presented using the median and interquartile range, a corresponding data transformation was performed. When outcomes were measured at different times, the last available measurement for both groups was used.

Relative risk was used for dichotomous outcomes, and for quantitative outcomes, the mean difference (MD) or the standardized mean difference (SMD) was employed. Outcomes not included in the meta-analysis due to the number or variability of the reports—namely, duration of the AOM clinical episode, hospitalizations, incidence of upper respiratory tract infections, and adverse events—were synthesized qualitatively to present their findings. Subgroup analyses were conducted based on treatment duration, type of probiotic strain, number of strains in the product supplied, age group, and co-interventions whenever these were applied in both the intervention and control arms. Sensitivity analyses were also performed.

Statistical heterogeneity was assessed using the I^2^ statistic. High heterogeneity was considered when the I^2^ value was equal to or greater than 50%. Fixed-effects models were used when I^2^ was less than 50%, and random-effects models were employed when I^2^ exceeded 50%. In both scenarios, the inverse variance method was used. In cases where multiple probiotic strains were used, regardless of heterogeneity, the random-effects model was chosen due to the expected variations in outcomes. A *p* value of <0.05 was considered statistically significant. Finally, publication bias was investigated by constructing funnel plots and performing Egger’s test using the Jamovi 2.3.28^®^ software.

## 3. Results

### 3.1. Studies Identified for the Review

**Figure 1:**
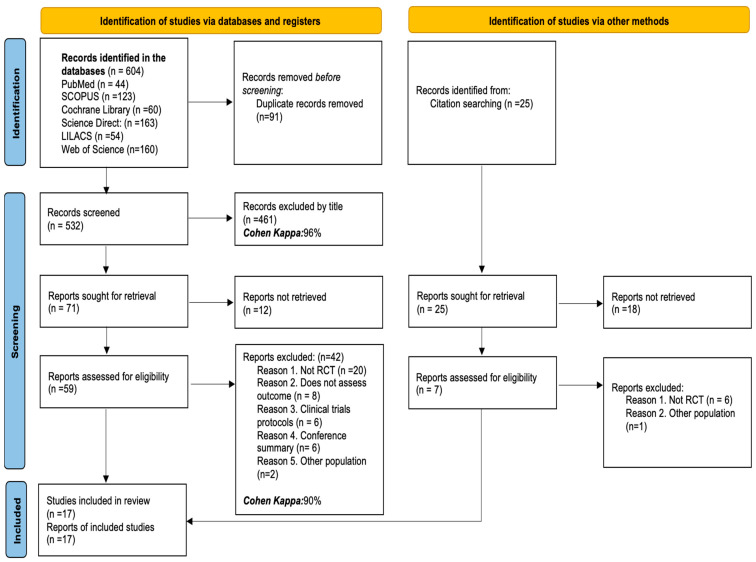
PRISMA flow diagram of studies selected for the review.

After conducting the search in the selected databases for this review, 604 initial records were identified. After removing 91 duplicate records, 532 articles remained for preliminary evaluation. Of these, 461 were discarded after reviewing the titles (Cohen Kappa: 96%). Next, 71 reports were selected for review, although 12 could not be retrieved. A total of 59 articles were evaluated in full text, of which 42 were excluded for the following reasons: twenty were not randomized controlled trials, eight did not address the outcomes defined in the review, six were clinical trial protocols, six were conference abstracts, and two analyzed a different population. As a result, 17 studies were included in the systematic review (Cohen Kappa: 90%). Moreover, 25 additional records were identified through citation searching. Of these, eighteen were not available for analysis, and seven articles were evaluated in full text, of which six were discarded for not being RCTs and one for focusing on a different population. Finally, the review incorporated a total of 17 studies. See details in Figure 1.

### 3.2. Characteristics of the Studies Included in the Review

Seventeen randomized controlled trials [34,35,36,37,38,39,40,41,42,43,44,45,46,47,48,49,50] conducted between 2004 and 2024 in seven European countries were analyzed, with Finland and Italy being the most frequent settings, with six and five studies, respectively. The study population consisted mainly of healthy children (82.3%), with ages ranging from the first month of life to 10 years. Three investigations focused on children at risk of AOM or with a history of disease recurrence.Most studies were conducted in daycare settings (9/17), followed by outpatient areas (7/17). The most evaluated outcomes included the development of AOM, present in 100% of the studies, followed by intervention-related adverse events (65%) and antibiotic requirement (53%). Additionally, four more studies addressed disease recurrence, and two investigations examined the time to the first AOM episode.

The temporal analysis revealed that more recent studies, such as those by Sarlin et al. [34] and Garaiova et al. [35], expanded the traditional outcomes by including hospitalizations, the incidence of upper respiratory infections, and temporal parameters such as the time to the first AOM episode. In contrast, previous research primarily focused on more basic clinical outcomes, such as the development of AOM. For further details, see Table 1.

### 3.3. Strains Administered for the Prevention or Treatment of AOM

In the studies included in the review, a total of 16 specific probiotic strains were identified, belonging to the genera *Lactobacillus*, *Bifidobacterium*, *Streptococcus*, and other bacteria. The genus *Lactobacillus* was the most represented, with seven strains (43.8%), notably *L. rhamnosus* with four variants (*LPR CGMCC 1.3824*, *GG ATCC 53103*, *LC 705,* and *LB21*), as well as *L. acidophilus* (*CUL21* and *CUL60*) and *L. paracasei* (*CBA L74*). Meanwhile, species from the genus *Bifidobacterium* represented four strains, including *B. breve* 99, *B. lactis* Bb-12, *B. bifidum* CUL20, and *B. animalis subsp. lactis* CUL34. These strains stand out for their immunomodulatory properties.

Similarly, the genus *Streptococcus* included four strains, among which *S. salivarius* presented three variants (*K12*, *24SMB*, and *DSM 13084*), and *S. thermophilus* NCC 2496 was added. These strains play a relevant role in respiratory and oral health, with *S. salivarius* K12 being one of the most studied for its potential capacity to prevent upper respiratory tract infections. Finally, a single strain from a different bacterial genus was reported (*Propionibacterium freudenreichii* shermanii JS). Overall, the strains of *Lactobacillus* and *Bifidobacterium* accounted for more than two-thirds of the total, indicating a greater preference in the studies for these bacteria, while the strains of *Streptococcus* and other species complemented the probiotic approach. This finding suggests a predominant interest in strains that have demonstrated more robust efficacy in immune modulation and the prevention of respiratory infections in children. See Table 2.

### 3.4. Characteristics of the Population and the Applied Intervention

The 17 studies included had a total of 4327 patients, of whom 2238 belonged to the intervention group and 2089 to the control group. The sample size of individual studies varied between 32 and 413 patients in the intervention groups and between 35 and 414 patients in the control groups. The proportion of male patients ranged between 43% and 57% in the studies that reported this data. The mean age of the participants ranged from less than 1 year to 6.6 years. Of the investigations included, 82% administered probiotics, 12% synbiotics, and the remainder prebiotics; see Table 3.

The probiotic, prebiotic, or synbiotic products were mainly administered orally in different presentations. It was identified that powdered milk was the most frequently used form, evaluated in six studies. This was followed by chewable tablets, which were analyzed in two studies, and tablets, which were present in four studies. Fermented milk was examined in two studies, while powder, nasal spray, and capsule were each considered in one study. Additionally, the treatment duration varied between 1 and 21 months, with 3 months being the most common intervention duration. Regarding the dosage of the products used, the number of colony-forming units per dose ranged between 1 × 10^7^ and 1 × 10^10^, depending on the strain and formulation. The most commonly used strains were *L. rhamnosus* GG (*n* = 6), *S. salivarius* K12 (*n* = 5), and *B. lactis* Bb-12 (*n* = 3). See Table 3.

### 3.5. Result of Risk of Bias Assessment

**Figure 2:**
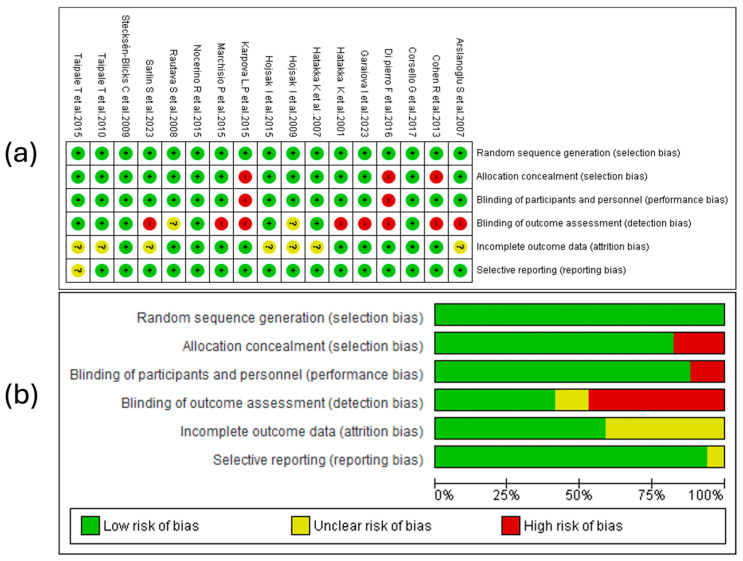
Risk of bias assessment of the studies included in the review [34,35,36,37,38,39,40,41,42,43,44,45,46,47,48,49,50]. (**a**) The symbol “+” denotes a low risk of bias, “?” indicates an unclear risk of bias, and “−” represents a high risk of bias. The colors associated with each symbol are green for low risk, yellow for unclear risk, and red for high risk. (**b**) A summary of the risk of bias identified after evaluating each study, showing the corresponding percentages for each bias domain.

The risk of bias analysis of the studies was conducted, considering different criteria, as shown in Figure 2. The findings from the evaluation performed with RevMan 5.4^®^ (accessed on 7–15 February 2025) are reported below.

#### 3.5.1. Random Sequence Generation

In all studies included in the review, the random sequence generation was assessed as a low risk. This indicates that the randomization methods were adequately described and applied in most of the evaluated clinical trials. The absence of evaluations indicating unclear or high risk in this domain reinforces the internal validity of the reported results.

#### 3.5.2. Allocation Concealment

The majority of the evaluated studies (14 out of 17) adequately concealed the allocation of participants to the different study groups [34,35,36,37,39,40,41,44,45,46,47,48,49,50]. In contrast, the studies by Cohen R et al. [43], Di Pierro F et al. [38], and Karpova L et al. [42] were rated as having a high risk of bias. Although the proportion of RCTs with allocation concealment issues was only 17%, this disparity in reporting quality highlights the need for future clinical trials to thoroughly document their allocation procedures according to recognized methodological standards to minimize selection bias and reinforce internal validity.

#### 3.5.3. Blinding of Participants and Personnel

In the assessment of the risk of bias associated with blinding of participants and personnel, 88% of the analyzed studies presented a low risk, indicating that appropriate methodological strategies were implemented to minimize expectancy effects. However, the studies by Di Pierro et al. [38] and Karpova L et al. [42] were classified as having a high risk of bias, suggesting the absence of effective measures to ensure that neither the participants nor the research personnel were aware of the intervention allocation.

The lack of blinding in these studies may have introduced performance bias, affecting aspects such as treatment adherence and perception of disease recurrence, thus compromising the internal validity of the findings. In trials on probiotics, prebiotics, and synbiotics for acute otitis media, where outcomes include subjective variables such as the duration of each AOM episode, this bias can exaggerate treatment effects. These results underscore the importance of adopting rigorous methodological strategies, such as using indistinguishable placebos and implementing double-blinding training, to ensure the validity and reproducibility of the scientific evidence.

#### 3.5.4. Blinding of Outcome Assessment

The risk of bias in the blinding of outcome assessment was classified as high in the studies by Arslanoglu S et al. [49], Cohen R et al. [43], Di Pierro F et al. [38], Garaiova I et al. [35], Hatakka k et al. [50], Karpova L et al. [42], Marchisio P et al. [40], and Salin et al. [34], indicating the absence of adequate methodological strategies to avoid subjective influences in the evaluation of clinical outcomes. In the studies by Hojsak I et al. [44] and Rautava S et al. [47], the risk was considered unclear, reflecting insufficient information regarding the implementation of blinding in outcome assessment. In contrast, the remaining studies were classified as having a low risk of bias, suggesting greater methodological rigor in minimizing detection bias. This underscores the need to improve methodological quality in future investigations by ensuring that outcome assessors are unaware of treatment allocation to obtain more reliable results.

#### 3.5.5. Incomplete Outcome Data

Regarding the handling of incomplete data, seven of the seventeen evaluated studies, including those by Arslanoglu S et al. [49], Hatakka K et al. [48], Hojsak I et al. [44], Hojsak I et al. [37], Sarlin S et al. [34], Taipale T et al. [45], and Taipale T et al. [39], presented an unclear risk of bias. This indicates a lack of sufficient information to determine whether data exclusion or loss may have influenced the results.

On the other hand, 58.8% of the analyzed studies showed a low risk of bias in this domain, suggesting that they implemented adequate strategies to minimize the impact of missing data in their analyses. The proper management of incomplete data in the majority of the reviewed studies strengthens the validity of their conclusions. However, the presence of uncertainty in a considerable proportion of studies highlights the need for more transparent and detailed reporting in future investigations.

#### 3.5.6. Selective Reporting

Regarding selective reporting bias, 16 of the 17 studies included in the review showed a low risk, indicating that outcomes were reported completely without evidence of omission of relevant results. This finding suggests adequate transparency in data communication, reinforcing the validity of the conclusions obtained. However, the study by Taipale T et al. [39] presented an unclear risk of bias in this domain, implying that there was not enough information to determine whether there was partial selection of the reported outcomes. The lack of clarity in this case highlights the importance of ensuring more rigorous reporting strategies in future research.

#### 3.5.7. Summary of Risk of Bias

In summary, the methodological analysis showed that random sequence generation presented low risk in 100% of the studies. However, 17.6% showed a high risk in allocation concealment, which could affect randomization. Blinding of participants and personnel was adequate in most cases, with only 11.8% at high risk, while outcome assessment showed greater vulnerability, with 50% at high risk. Regarding incomplete data, 41.2% presented an unclear risk, although the remainder was adequately handled. Selective reporting was the least problematic, with only one study at an unclear risk (5.9%), indicating transparency in most cases. These findings highlight the need to improve blinding in outcome assessment and optimize the management of missing data to strengthen the validity of the evidence. See Figure 2, part b.

### 3.6. Qualitative Synthesis of the Scientific Evidence

#### 3.6.1. Duration of AOM Episodes

Of the seventeen studies included in the review, two evaluated the duration of AOM episodes. In this regard, Hatakka et al. [48], in a population of 309 children randomly distributed to consume a multi-strain probiotic (*n* = 155) or placebo (*n* = 154) for a period of 24 weeks, found that the median duration of AOM episodes was 5.6 days in the intervention group and 6 days in the placebo group, suggesting no significant difference between the treatment arms. In contrast, Stecksén-Blicks C et al. [46] evidenced a shorter duration of AOM episodes in patients treated with a probiotic preparation. After studying a sample of 248 children, of whom 133 received 150 mL of milk supplemented with *L. rhamnosus* LB21 for 21 months, they found that the intervention group had a duration of 0.5 ± 2.2 days versus 1.0 ± 2.7 days in the control group (*p* = 0.003).

#### 3.6.2. Hospitalizations

Only one study reported data on hospitalizations in patients with acute otitis media [34]. In this study, eligible participants were randomly assigned to a daily regimen of *S. salivarius* K12 or placebo for a period of six months; the daily dose and mode of administration were standardized according to age, administering the probiotic as a soluble powder for children under 3 years and as a chewable tablet for older children. Regarding the outcome, no significant differences were found in the proportion of children with at least one medical visit due to an acute illness or in the proportion of children hospitalized for acute respiratory symptoms.

#### 3.6.3. Incidence of Upper Respiratory Tract Infections

Three of the 17 studies in the review evaluated the incidence of upper respiratory tract infections in the participants. In this regard, Garaiova I et al. [35] conducted an investigation to establish whether the combination of a multi-strain probiotic product associated with vitamin C had an impact on antibiotic use and the presence of upper and lower respiratory complications. After analyzing 204 patients, nearly evenly divided between the study arms, they found no statistically or clinically relevant difference in the proportion of children who developed an upper respiratory tract infection (probiotic 33.98% vs. placebo 37.6%). This finding is consistent with that of Hatakka K et al. [48] in 309 children who received a daily capsule of a probiotic composed of *L. rhamnosus* GG and LC705, *B. breve* 99, and *P. freudenreichii* JS. After the follow-up period, they concluded that the average number of upper respiratory tract infections was similar in both groups (probiotic 4.3 ± 1.4 vs. placebo 4.6 ± 1.4, *p* > 0.05).

In contrast, Arslanoglu S et al. [49], in a population of 204 patients (102 receiving a prebiotic preparation composed of galactooligosaccharides/fructooligosaccharides for 6 months), found that the incidence of upper respiratory tract infections was significantly lower in the intervention group than in the control group, reaching approximately 10% (*p* < 0.05).

#### 3.6.4. Adverse Effects

Most of the studies included did not report adverse events associated with the use of probiotics, including the strains *S. salivarius* K12, *L. rhamnosus* GG, *B. lactis*, *L. reuteri*, and *L. casei*, compared to placebo. Six studies did not identify adverse effects in any of the participants, including those by de Corsello et al. [36], Di Pierro et al. [38], Hojsak et al. [37], Nocerino et al. [41], Stecksén-Blicks et al. [46], and Hatakka et al. [50].

In studies that reported adverse events, these were predominantly mild and self-limited. Taipale et al. [39,45] described the presence of gastrointestinal discomfort and dermatological reactions, while Rautava et al. [47] reported vomiting and flatulence in some participants. Cohen et al. [43] documented effects such as lack of appetite, regurgitation, dry skin, diarrhea, constipation, and abdominal pain, although most were not attributed to the treatment.

A relevant finding was that of Marchisio et al. [40], who reported a higher frequency of adverse events in the intervention group (42%) compared to the control group (15%), including symptoms such as sneezing, itching, cough, nasal congestion, epistaxis, and rhinorrhea, although without evidence of systemic or severe adverse events. In general, no study reported serious adverse effects attributable to the administration of probiotics, which supports their safety profile in the evaluated pediatric population. See Table 4.

### 3.7. Meta-Analysis

A meta-analysis was performed for four outcomes based on the studies by Sarlin S et al. [34], Garaiova I et al. [35], Corsello G et al. [36], Hojsak I et al. [37], Di Pierro F et al. [38], Taipale T et al. [39], Marchisio P et al. [40], Nocerino R et al. [41], Karpova E et al. [42], Cohen R et al. [43], Hojsak I et al. [44], Taipale T et al. [45], Rautava S et al. [47], Hatakka K et al. [48], Arslanoglu S et al. [49], and Hatakka K et al. [50].

#### 3.7.1. Result of the Assessment of the Quality of Evidence

Among the 17 evaluated studies, 15 obtained scores of 4 points or more, indicating that most met the key methodological criteria, including randomization, reporting of dropouts, proper execution of the allocation procedure, and clear presentation of the inclusion and exclusion criteria. Six studies [35,36,40,41,43,50] achieved the maximum score of 5 points, reflecting high methodological quality. However, the implementation of the double-blind design was inconsistent, with two studies [38,42] scoring ≤ 3 points due to the absence of this control. The lack of blinding in these trials could be related to limitations in protocol implementation or the absence of strict measures to minimize performance and detection bias. Despite these differences, the majority of the studies demonstrated adequate methodological rigor. For further details, see Table 5.

#### 3.7.2. Time to AOM Episode

**Figure 3:**
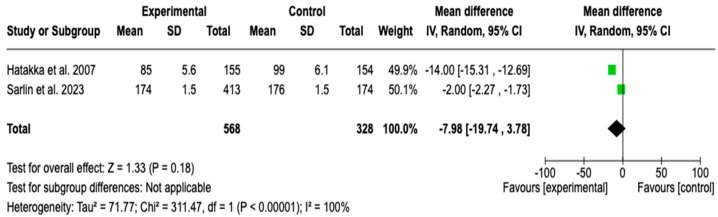
Forest plots of the effect of adjunctive therapy with probiotics, prebiotics, or synbiotics on the time to AOM episode [34,48].

Two studies [34,50] with a total of 896 patients evaluated the effect of probiotic supplementation on the time to acute otitis media. A chi-square of 311.47 (*p* < 0.00001) and an I^2^ = 100% were observed. The meta-analysis showed that probiotic supplementation did not have a significant effect on the time to AOM (MD: −7.98, 95% CI: −19.74 to 3.78; *p* = 0.18). Although heterogeneity was high, the limited number of studies for this outcome did not allow for subgroup or sensitivity analyses, restricting the exploration of potential sources of variability in the results. See Figure 3.

#### 3.7.3. Recurrence of Acute Otitis Media

**Figure 4:**
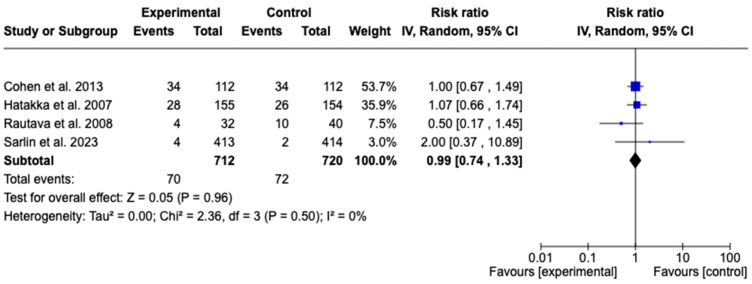
Forest plots of the effect of adjunctive therapy with probiotics, prebiotics, or synbiotics on AOM recurrence [34,43,47,48].

On the other hand, the studies by Cohen R et al. [43], Hatakka K et al. [48], Rautava S et al. [47], and Sarlin S et al. [34] were included in the analysis of AOM recurrence, with a total of 1432 patients. The meta-analysis did not show a statistically significant effect (RR: 0.99; 95% CI: 0.74–1.33; *p* = 0.96). Heterogeneity was null (I^2^ = 0%), indicating high consistency among the studies; therefore, no subgroup or sensitivity analyses were conducted. The findings do not support the use of prebiotic, probiotic, or synbiotic supplementation to reduce the risk of AOM recurrence. See Figure 4.

#### 3.7.4. Antibiotic Requirement for Acute Otitis Media

**Figure 5:**
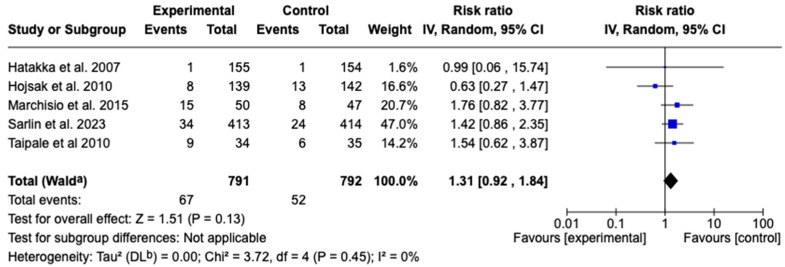
Forest plots of the effect of adjunctive therapy with probiotics, prebiotics, or synbiotics on the use of antibiotics for the treatment of AOM [34,40,44,45,48].

Five studies [34,40,44,45,48] with a total of 1583 patients evaluated the effect of probiotic supplementation on the antibiotic requirement for acute otitis media. A chi-square of 3.72 (*p* = 0.45) and an I^2^ = 0% were observed, indicating an absence of heterogeneity. The meta-analysis did not show a significant effect of probiotic supplementation on the antibiotic requirement for acute otitis media (RR: 1.31; 95% CI: 0.92 to 1.84; *p* = 0.13). Given the heterogeneity, subgroup or sensitivity analyses were not justified. See Figure 5.

#### 3.7.5. Incidence of Acute Otitis Media

**Figure 6:**
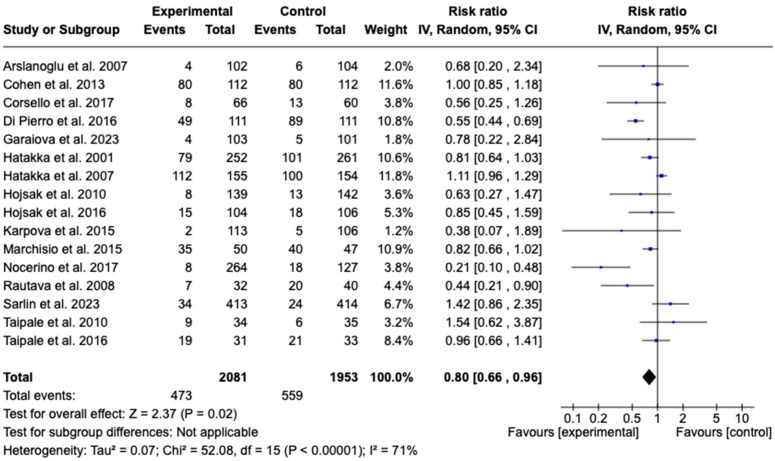
Forest plots of the effect of adjunctive therapy with probiotics, prebiotics, or synbiotics on the incidence of acute otitis media [34,35,36,37,38,39,40,41,42,43,44,45,47,48,49,50].

Sixteen studies [34,35,36,37,38,39,40,41,42,43,44,45,47,48,49,50] with a total of 4034 patients evaluated the effect of probiotic supplementation on the incidence of acute otitis media. A chi-square of 52.08 (*p* < 0.00001) and an I^2^ = 71% were observed. The meta-analysis showed that probiotic supplementation significantly reduced the incidence of acute otitis media (RR: 0.80; 95% CI: 0.66 to 0.96; *p* = 0.02). See Figure 6.

**Figure 7:**
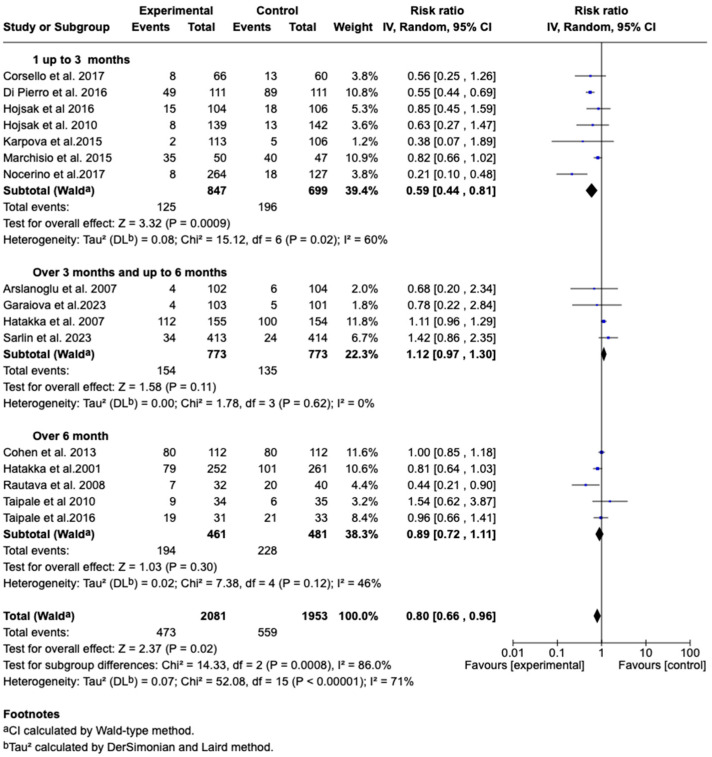
Forest plots of the subgroup analysis on the effect of probiotics, prebiotics, or synbiotics on the incidence of AOM according to treatment duration [34,35,36,37,38,39,40,41,42,43,44,45,47,48,49,50].

Subgroup analysis according to treatment duration showed that in the group with treatment up to three months (*n* = 847 patients), the combined effect was RR: 0.59 (95% CI: 0.44–0.81; *p* = 0.0009), with 60% heterogeneity. In the group with treatment between more than three and up to six months (*n* = 773 patients), the effect was RR: 1.12 (95% CI: 0.97–1.30; *p* = 0.11), with no evidence of heterogeneity (I^2^ = 0%). Finally, in the group with treatment longer than six months (*n* = 461 patients), the effect was RR: 0.89 (95% CI: 0.72–1.11; *p* = 0.30), with moderate heterogeneity (I^2^ = 46%). Overall, the effect of the treatment was different between the subgroups (*p* = 0.0008), with an overall heterogeneity of 71%. This suggests that the treatment duration could influence the observed effectiveness. See Figure 7.

**Figure 8:**
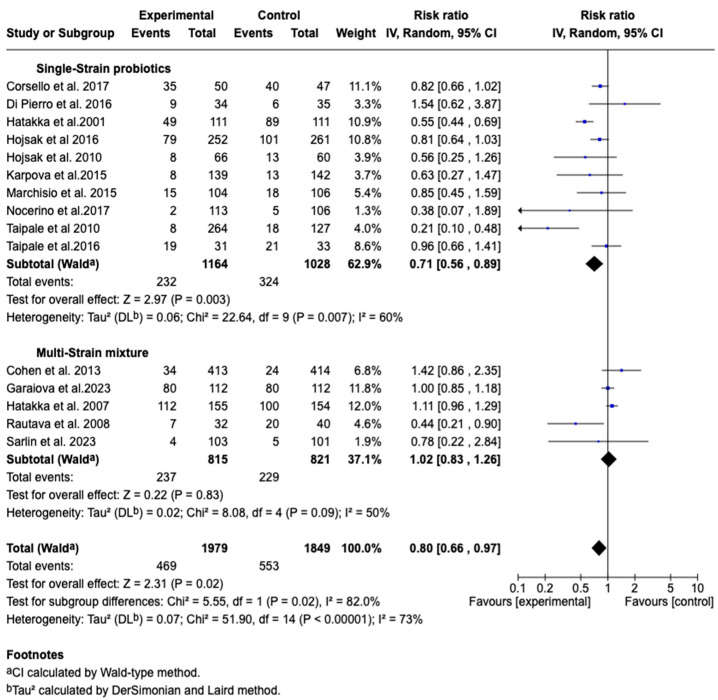
Forest plots of the subgroup analysis on the effect of probiotics, prebiotics, or synbiotics on the incidence of AOM according to the number of strains included in the product [34,35,36,37,38,39,40,41,42,43,44,45,47,48,49,50].

Furthermore, in the subgroup analysis by type of probiotic, it was shown that in the group receiving a single strain (*n* = 1164 patients), the combined effect was RR: 0.71 (95% CI: 0.56–0.89; *p* = 0.003), with 60% heterogeneity. In the group receiving a mixture of multiple strains (*n* = 815 patients), the effect was RR: 1.02 (95% CI: 0.83–1.26; *p* = 0.83), with 50% heterogeneity. Overall, the effect of the treatment was significantly different between the subgroups (*p* = 0.02), with an overall heterogeneity of 73%. This suggests that single-strain probiotics might be more effective compared to multi-strain mixtures in the prevention or treatment of otitis media. See Figure 8.

**Figure 9:**
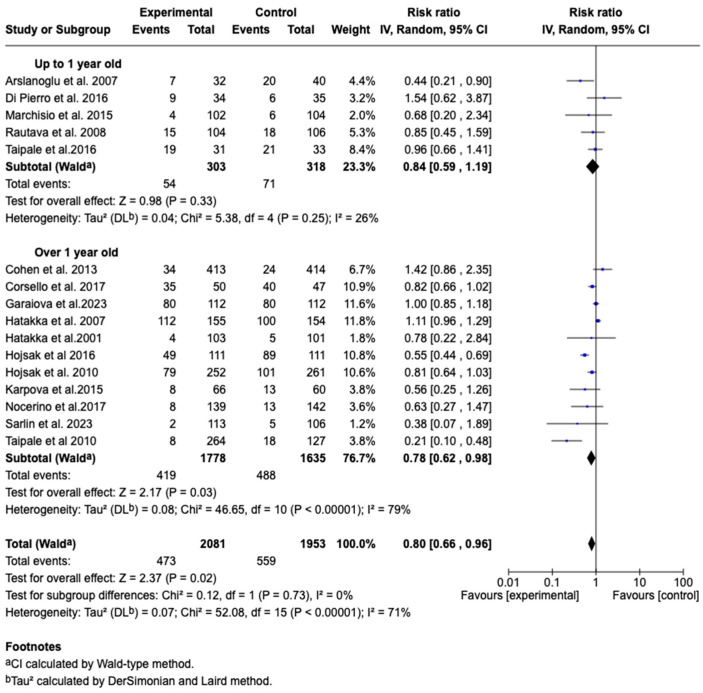
Forest plots of the subgroup analysis on the effect of probiotics, prebiotics, or synbiotics on the incidence of AOM according to the age group of the patients [34,35,36,37,38,39,40,41,42,43,44,45,47,48,49,50].

Subgroup analysis according to the age of the patients showed that in the group up to 1 year old (*n* = 303 patients), the combined effect was RR: 0.84 (95% CI: 0.59–1.19; *p* = 0.33), with 26% heterogeneity. In the group older than 1 year (*n* = 1778 patients), the effect was RR: 0.78 (95% CI: 0.62–0.98; *p* = 0.03), with 79% heterogeneity. Overall, the effect of the treatment was not significantly different between the subgroups (*p* = 0.73), with an overall heterogeneity of 71%. This suggests that, although the effect appears clearer in those older than 1 year, age may not be a determining factor in treatment efficacy. However, the high heterogeneity in this group indicates that other influencing factors may exist. See Figure 9.

In order to assess the stability of the results and the impact of studies with limited methodological quality on the estimation of the treatment effect, a sensitivity analysis was performed excluding the studies by Di Pierro et al. [38] and Karpova et al. [42]. This was considered because they obtained a score of 3 in the quality assessment, indicating a high risk of bias due to deficiencies in the blinding and randomization processes. After performing the analysis, their exclusion did not affect the results, suggesting that the estimation of the treatment effect does not change significantly (see Supplementary Materials, Figures S1 and S2).

#### 3.7.6. Publication Bias

The evaluation of publication bias was not feasible for the outcomes “time to the first AOM episode”, “recurrence of acute otitis media”, and “antibiotic requirement for AOM” due to the insufficient number of studies in each case. However, the analysis was performed for the outcome “incidence of AOM”.

**Figure 10:**
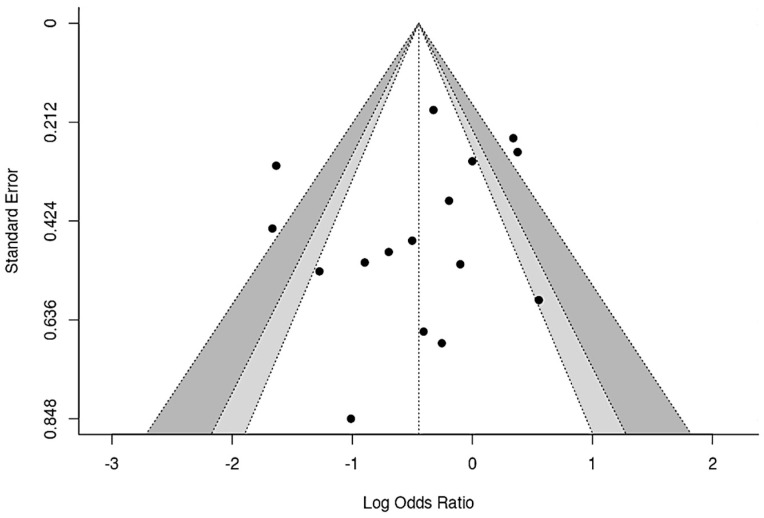
Funnel plots on publication bias regarding the effect of probiotics, prebiotics, or synbiotics on the incidence of AOM [34,35,36,37,38,39,40,41,42,43,44,45,47,48,49,50].

To evaluate publication bias, a funnel plot was constructed (Figure 10), in which slight asymmetry of the studies around the estimated effect was observed. However, Egger’s test yielded a value of −1.470 with a *p*-value of 0.142, indicating that the correlation between the standard error and the estimated effect is not statistically significant. This suggests that the evidence of publication bias is not conclusive and that the observed asymmetry in the plot may be due to variability among the included studies rather than systematic publication bias.

## 4. Discussion

### 4.1. Main Findings

The primary objective of this systematic review and meta-analysis was to evaluate the effectiveness of probiotic, prebiotic, or synbiotic supplementation in the prevention and treatment of acute otitis media in pediatric populations. A total of 17 randomized controlled trials were included, with over 4000 patients evaluated across diverse clinical settings. The meta-analysis revealed a statistically significant reduction in the overall incidence of AOM in children receiving supplementation (RR: 0.80; 95% CI: 0.66–0.96; *p* = 0.02). This finding suggests that, on balance, these supplements may help reduce the burden of AOM in children.

However, while the reduction in incidence is promising, other key clinical endpoints did not reach statistical significance. For example, analyses of the time to the first AOM episode and the recurrence of AOM showed no significant differences between the intervention and control groups. Similarly, the requirement for antibiotics during AOM episodes was not significantly altered by supplementation (RR: 1.31; 95% CI: 0.92–1.84; *p* = 0.13). These null findings underscore that while probiotics, prebiotics, or synbiotics may have a role in lowering AOM occurrence, their influence on modifying disease course or severity appears limited.

Subgroup analyses provided further insight. The effectiveness of supplementation was influenced by treatment duration, with interventions of up to three months yielding a more pronounced effect (RR: 0.59; 95% CI: 0.44–0.81; *p* = 0.0009) compared to longer regimens. Moreover, products formulated with a single probiotic strain demonstrated a significant benefit (RR: 0.71; 95% CI: 0.56–0.89; *p* = 0.003), whereas multi-strain mixtures did not produce a significant effect. These subgroup findings suggest that both the duration of treatment and the specificity of the probiotic formulation are critical variables in determining clinical outcomes.

### 4.2. Comparison with Previous Studies

The methodological quality of the included trials was assessed using the Jadad scale, and robust statistical tools were employed to analyze heterogeneity and conduct subgroup analyses. This methodological rigor clearly distinguishes this study from previous reviews, providing a comprehensive perspective on the use of probiotics for respiratory infections across various populations and contexts.

Scott et al., 2019 [29] present a design similar to this study, as they also conducted a meta-analysis of 17 RCTs but focused exclusively on the prevention of AOM in children. Using Cochrane methodology and the GRADE system to assess evidence certainty, this review explicitly distinguishes between children predisposed and not predisposed to AOM, finding that probiotics significantly reduce incidence only in non-predisposed children (RR: 0.64), while no significant benefit is observed in predisposed children (RR: 0.97). This reinforces the notion proposed in this study about the importance of considering individual patient characteristics when evaluating the effectiveness of these interventions.

On the other hand, Zhang et al., 2025 [51] adopt a broader perspective, reviewing recent advances regarding the role of probiotics in preventing and treating recurrent respiratory infections, including AOM. Although it is a narrative review, Zhang et al. [51] delve into the immunological mechanisms of the gut-lung axis, highlighting critical factors such as dose, treatment duration, and probiotic strain specificity. Thus, Zhang et al.’s [51] review complements the quantitative findings of the current study, providing a physiological basis for the observed heterogeneity in clinical outcomes.

Similarly, Laursen and Hojsak, 2018 [52] focus their analysis on children attending daycare, a population particularly susceptible to respiratory infections such as AOM. Their review emphasizes the impact of the daycare environment on infection incidence and highlights the importance of strain-specific analysis, particularly *Lactobacillus rhamnosus* GG. Their conclusions underscore the need for precise and targeted interventions, also identified as essential by the current study.

Esposito et al., 2014 [53] provide a cautious mini-review on probiotic use for AOM prevention, emphasizing that nasal formulations might be superior to oral ones in effectively colonizing the nasopharynx and preventing infection. This point highlights the clinical relevance of the administration route, also noted in the current study’s analysis.

Coleman et al., 2019 [25] offer a narrative review presenting historical and contemporary perspectives on probiotic use in AOM, highlighting a shift towards more localized strategies (intranasal administration) and emphasizing that “niche-specific” strains from the nasopharynx itself could provide more effective protection. This approach aligns with findings from the current study, suggesting greater attention to strain selection and the administration route to optimize clinical outcomes.

Chen et al., 2020 [54] conclude that the overall current evidence supporting probiotic use for AOM prevention is limited and of moderate to low quality, especially regarding oral formulations. This review highlights the need for future rigorous and homogeneous studies, corroborating the current study’s conclusion regarding significant heterogeneity and the importance of identifying efficacy moderators.

Finally, Zhao et al., 2022 [55] broaden the scope by evaluating probiotics for preventing acute upper respiratory tract infections in children, adults, and older adults. Employing rigorous methods and analyses based on the GRADE system, this review finds significant reductions in infection incidence and duration, as well as antibiotic use, supporting the general utility of probiotics in respiratory infections. Although Zhao et al. [55] examine a broader spectrum than the current study, both reviews agree that efficacy largely depends on dosage, treatment duration, and strain specificity.

These comparisons highlight that although evidence supports a moderate benefit of probiotics in preventing AOM and related respiratory infections, critical questions remain regarding strain selection, optimal administration route, and ideal treatment protocols. This study provide a robust quantitative synthesis specific to the pediatric context, offering strong data to guide future research and clinical applications, complemented by perspectives from the studies cited above.

### 4.3. Possible Mechanisms of Action

The observed reduction in the incidence of AOM following probiotic administration can be explained by several interrelated mechanisms that act both locally and systemically. Firstly, these microorganisms modulate the host immune response by regulating the production of inflammatory cytokines and enhancing mucosal barrier function. This study emphasizes that the action of probiotics leads to a more controlled immune response, as they stimulate the production of anti-inflammatory mediators and reduce the proinflammatory environment, which in turn hinders bacterial colonization in the middle ear.

Additionally, Godur et al., 2023 [56] have provided evidence of the capacity of certain probiotics to convert nondigestible carbohydrates into short-chain fatty acids (SCFAs). These metabolites are essential for maintaining the integrity of the mucosal barrier in the respiratory tract, reducing its permeability, and preventing pathogenic microorganisms from invading the middle ear. The stability of this barrier is crucial for infection prevention, as an intact mucosa limits the translocation of bacteria that might otherwise trigger AOM.

Furthermore, Peng et al., 2024 [57] have demonstrated that *Streptococcus salivarius* K12, a strain with a high capacity for oral colonization, produces bacteriocins and other antimicrobial substances (BLIS) that directly inhibit the growth of pathogenic bacteria. This competition for adhesion sites in the nasopharynx prevents colonization by pathogens such as *Streptococcus pneumoniae*, which is associated with a lower incidence of AOM. The effectiveness observed with single-strain formulations, such as that of *S. salivarius* K12, suggests that these strains may offer a more specific and potent action compared to multi-strain formulations, in which immunomodulatory effects could be diluted or antagonized.

Moreover, Zhao et al., 2022 [55] emphasize that the modulation of the gut–lung axis represents another relevant mechanism. Oral administration of probiotics not only improves the composition of the intestinal microbiota but also induces the production of SCFAs, which reinforce mucosal integrity and regulate inflammation systemically. This systemic effect creates an environment that is less conducive to pathogen proliferation, thereby contributing to a reduced likelihood of developing infections such as AOM.

Complementary studies, such as those by Laws et al., 2021 [58], have shown that ingestion of *S. salivarius* BLIS K12 can induce immunomodulatory responses, reflected in an increased frequency of regulatory T cells (Tregs) and elevated IL-10 concentrations, which favor an anti-inflammatory environment. The concurrent decrease in IL-6 observed in these studies also appears to support the expansion of Tregs, as IL-6 is known to antagonize their proliferation. In addition, Zhang et al., 2025 [51] have highlighted the importance of the gut–lung axis, indicating that modulation of the intestinal microbiota through probiotics influences the immune function of the respiratory tract by reinforcing the mucosal barrier and attenuating the inflammatory response.

The integration of these mechanisms, which include competition for colonization sites, production of bacteriocins, and regulation of the immune response through anti-inflammatory mediators, supports the hypothesis that careful selection of specific strains and optimization of the administration route are key factors in achieving an optimal preventive effect against AOM. Each of these studies underscores the importance of evaluating not only the effectiveness of probiotics but also the underlying mechanisms that enable their beneficial action in preventing middle ear infections [59,60,61].

### 4.4. Limitations of the Included Studies

When analyzing the 17 studies included in this systematic review, several important limitations related to study design, population characterization, heterogeneity, and effect size emerged and merit discussion.

Although many studies implemented robust methods for random sequence generation, notable methodological shortcomings were present in other areas. For instance, approximately 17.6% of studies exhibited a high risk of bias due to inadequate allocation concealment, raising concerns about potential selection bias. Furthermore, nearly half of the studies lacked sufficient blinding of outcome assessors. Given the subjective nature of outcomes such as the duration and severity of AOM, inadequate blinding may have led to biased estimations of treatment effectiveness, either exaggerating or diminishing actual effects.

Another critical limitation pertains to managing incomplete outcome data. While more than half of the studies effectively addressed dropout rates and missing data, a significant proportion provided insufficient detail to determine whether data exclusions influenced the overall results. Such a lack of transparency, coupled with varied reporting methods, complicates evidence synthesis and limits comparability across studies.

Substantial heterogeneity among studies further weakens the strength of the pooled conclusions. Treatment durations ranged widely from 1 to 21 months, and probiotic dosages varied considerably, from 1 × 10^7^ to 1 × 10^10^ colony-forming units. Additionally, the studies investigated different probiotic strains, each with distinct immunomodulatory and antimicrobial properties, and were conducted across diverse settings, including daycare centers, outpatient clinics, and hospitals. High I^2^ statistics for outcomes such as time to first AOM episode and overall AOM incidence indicate substantial variability, reducing the reliability and consistency of pooled effect estimates.

Characterization of the study populations posed additional challenges. Despite most participants being healthy children, considerable variation existed in age ranges (from infants to 10-year-olds) and risk profiles, with only a few studies explicitly targeting children with an elevated risk or recurrent AOM. Such heterogeneity in participant characteristics potentially dilutes observed effect sizes and limits the generalizability of findings to the broader pediatric population.

Lastly, although the meta-analysis demonstrated a statistically significant reduction in AOM incidence with probiotic supplementation, the effect size varied notably among individual studies. Differences in probiotic strains, dosage regimens, and modes of administration (oral versus nasal) likely contributed to these variations, underscoring the necessity of standardized protocols in future research.

Collectively, these limitations—including methodological flaws, inconsistent population characterization, significant heterogeneity, and variable effect sizes—highlight the pressing need for high-quality, standardized randomized controlled trials to accurately determine the efficacy of probiotics in preventing acute otitis media.

### 4.5. Clinical Implications

The evidence suggests that probiotic, prebiotic, or synbiotic supplementation can be considered a complementary strategy to reduce the incidence of AOM in children. The results are particularly encouraging for interventions with shorter durations and single-strain formulations. Nevertheless, the lack of significant impact on other clinically important outcomes, such as the duration of AOM episodes, recurrence rates, and antibiotic use, indicates that these supplements should not replace conventional treatment protocols. Instead, they may be integrated as part of a broader, multidisciplinary approach that includes established measures such as vaccination, appropriate antibiotic therapy, and supportive care, tailored to individual patient profiles.

### 4.6. Recommendations for Future Research

To refine our understanding of the role of probiotics in preventing and managing AOM, future studies should focus on conducting larger, multicenter RCTs with extended follow-up periods to enhance the robustness and generalizability of findings. It is essential to standardize intervention protocols, specifying probiotic strains, dosages, durations, and modes of administration to facilitate more reliable comparisons.

Additionally, incorporating advanced microbiome analysis techniques, such as metagenomics and 16S rRNA sequencing, could help clarify the underlying mechanisms through which probiotics influence the host immune response and microbial composition. Future research should also involve head-to-head comparisons between single-strain and multi-strain probiotic formulations to identify the most effective approaches [62,63].

Finally, expanding the range of clinical outcomes assessed, including quality of life, duration and severity of AOM episodes, and long-term respiratory health, will provide a more comprehensive evaluation of the therapeutic potential and limitations of probiotic interventions.

## 5. Conclusions

Supplementation with probiotics, prebiotics, or synbiotics is associated with a significant reduction in the incidence of AOM among children. However, no significant impact was observed on the time to the first episode, recurrence of the disease, or antibiotic use. These findings suggest that, particularly in short-term interventions using single-strain formulations, such strategies may serve as valuable adjuncts to conventional treatments, despite considerable heterogeneity in methods, dosages, and population characteristics across studies. Therefore, further controlled and standardized studies are necessary to confirm these results and precisely define optimal conditions for the clinical implementation of these interventions.

## Figures and Tables

**Table 1:** Characteristics of the studies included in the review.

Author and Year	Country	Design	Subjects	Setting	Evaluated Outcomes
Sarlin S et al., 2024 [34]	Finland	RCT	Healthy children at risk of AOM, between 1 to 6 years old	Daycares and schools	Development of AOM, time to the first AOM episode, disease recurrence, antibiotic requirement, hospitalizations, and incidence of upper respiratory tract infections.
Garaiova I et al., 2023 [35]	Slovakia	RCT	Healthy children between 3 and 10 years old	Outpatient and daycares	Development of AOM and incidence of upper respiratory tract infections.
Corsello G et al., 2017 [36]	Italy	RCT	Healthy children between 12 and 48 months old	Daycares	Development of AOM and adverse events.
Hojsak I et al., 2016 [37]	Croatia	RCT	Healthy children, between 1 and 6 months old	Daycares	Development of AOM and adverse events.
Di Pierro F et al., 2016 [38]	Italy	RCT	Healthy children around 3 years old	Daycares	Development of AOM and adverse events.
Taipale T et al., 2016 [39]	Finland	RCT	Healthy children from 1 month of age	Outpatient	Development of AOM and adverse events.
Marchisio P et al., 2015 [40]	Italy	RCT	Children with recurrent AOM, between 1 and 5 years old	Outpatient, Hospital	Development of AOM, antibiotic requirement, and adverse events.
Nocerino R et al., 2015 [41]	Italy	RCT	Healthy children between 12 and 48 months old	Outpatient	Development of AOM and adverse events.
Karpova E et al., 2015 [42]	Russia	RCT	Sick children, between 6 and 7 years old	Outpatient	Development of AOM.
Cohen R et al., 2013 [43]	France	RCT	Healthy children at risk of AOM, between 7 and 13 months old	Outpatient	Development of AOM, disease recurrence, antibiotic requirement, and adverse events.
Hojsak I et al., 2010 [44]	Croatia	RCT	Healthy children with an average age of 52 months	Daycares	Development of AOM and antibiotic requirement.
Taipale T et al., 2010 [45]	Finland	RCT	Healthy children from 1 month of birth	Outpatient	Development of AOM, antibiotic requirement, and adverse events.
Stecksén-Blicks C et al., 2009 [46]	Sweden	RCT	Healthy children between 1 and 5 years old	Daycare	Duration of the AOM episode, antibiotic requirement, and adverse events.
Rautava S et al., 2008 [47]	Finland	RCT	Healthy children requiring infant formula before 2 months of age	Outpatient	Development of AOM, disease recurrence, antibiotic requirement, and adverse events.
Hatakka K et al., 2007 [48]	Finland	RCT	Healthy children at risk of AOM, between 10 months and 6 years old	Daycares and primary care centers	Development of AOM, time to the first episode of the disease, disease recurrence, duration of the clinical episode, antibiotic requirement, and incidence of upper respiratory tract infections.
Arslanoglu S et al., 2007 [49]	Italy	RCT	Healthy children within the first 2 weeks of life	Outpatient	Development of AOM and incidence of upper respiratory tract infections.
Hatakka K et al., 2001 [50]	Finland	RCT	Healthy children at risk of AOM, between 1 and 6 years old	Daycares	Development of AOM, antibiotic requirement, and adverse events.

AOM = acute otitis media.

**Table 2:** Specific strains used as probiotics in the studies included in the review.

*Lactobacillus* spp.	*Bifidobacterium* spp.	*Streptococcus* spp.	Others
*L. rhamnosus* LPR CGMCC 1.3824	*B. breve* 99	*S. salivarius* K12	*P. freudenreichii* shermanii JS
*L. rhamnosus* GG ATCC 53103	*B. lactis* Bb-12	*S. salivarius* 24SMB	
*L. rhamnosus* LC 705	*B. bifidum* CUL20	*S. salivarius* DSM 13084	
*L. rhamnosus* LB21	*B. animalis subsp. lactis* CUL34	*S. thermophilus* NCC 2496	
*L. acidophilus* CUL21			
*L. acidophilus* CUL60			
*L. paracasei* CBA L74			

**Table 3:** Characteristics of the included population and the administered treatment.

Study	Patients (I/C)	% Male	Mean Age (Years)	Product (Type, Presentation, Strain, Dose, Route of Administration)	Treatment Duration (Months)	Conclusion
Sarlin S et al., 2024 [34]	I:413 C:414	52	4.1	Synbiotic. Soluble powder or chewable tablet. *S. salivarius* K12 1 × 10^9^ CFU, *L. rhamnosus* GG 1 × 10^8^ CFU, *P. freudenreichii* shermanii JS 1 × 10^8^ CFU, fructooligosaccharide 480 mg. Soluble powder or chewable tablet. Oral route.	6	The use of a product containing *S. salivarius* K12 did not reduce the occurrence of acute otitis media in the studied population.
Garaiova I et al., 2023 [35]	I:103 C:101	50.4	6.6	Probiotic. Chewable tablet. *L. acidophilus* CUL21 and CUL60, *B. bifidum* CUL20, *B. animalis subsp. lactis* CUL34. 1.25 × 10^10^ CFU. Oral route.	6	Supplementation with the probiotic combination used may reduce antibiotic requirements in pediatric patients.
Corsello G et al., 2017 [36]	I:66 C:60	57	2.7	Probiotic. Powdered cow’s milk. *L. paracasei* CBA L74. 5.9 × 10^9^ CFU. Oral route.	3	The use of fermented cow’s milk with *L. paracasei* may prevent some childhood infections.
Hojsak I et al., 2016 [37]	I:104 C:106	43	4.4	Probiotic. Powder. *B. animalis subsp. lactis* CUL34. 1 × 10^9^ CFU. Oral route.	3	The findings suggest that the administered strain does not prevent respiratory infections in the included children.
Di Pierro F et al., 2016 [38]	I:111 C:111	48	3	Probiotic. Tablets. *S. salivarius* K12. 1 × 10^9^ CFU. Oral route.	3	The administration of *S. salivarius* K12 was associated with a reduction in AOM episodes.
Taipale T et al., 2016 [39]	I:55 C:54	NS	<1 year	Probiotic. Tablet. *B. lactis* Bb-12. 1 × 10^10^ CFU. Oral route.	12	The administration of the supplied probiotic may reduce respiratory tract infections.
Marchisio P et al., 2015 [40]	I:50 C:47	NS	2.9	Probiotic. Nasal spray. *S. salivarius* 24SMB. 100 × 10^9^ CFU; 1 dose per nostril.	3	Probiotic supplementation with *S. salivarius* 24SMB via the intranasal route has the capacity to reduce the risk of AOM.
Nocerino R et al., 2015 [41]	I:264 C:127	51	2.6	Probiotic. Fermented milk. *L. paracasei* CBA L74. 5.9 × 10^9^ CFU. Oral route.	3	Fermented cow or rice milk with *L. paracasei* CBA L74 can prevent some common childhood infections.
Karpova E et al., 2015 [42]	I:113 C:106	NS	NS	Probiotic. Tablet. *S. salivarius* K12. Oral route.	1	The administered probiotic complex was associated with a lower frequency of otitis media in the studied patients.
Cohen R et al., 2013 [43]	I:112 C:112	NS	<1 year	Synbiotic. Milk. *S. salivarius* 2.5 × 10^7^ CFU, *S. thermophilus* 1 × 10^7^ CFU, *L. rhamnosus* 1 × 10^7^ CFU + Raftiline. Oral route.	12	The synbiotic administered in children did not reduce the risk of AOM, recurrent AOM, antibiotic use, or lower respiratory tract infections within 1 year.
Hojsak I et al., 2010 [44]	I:139 C:142	56	4.3	Probiotic. Fermented milk. *L. rhamnosus* GG 1 × 10^9^ CFU in 100 mL. Oral route.	3	The probiotic used in the study is recommended as a valid measure to reduce the risk of upper respiratory tract infections in children attending daycare.
Taipale T et al., 2010 [45]	I:34 C:35	NS	NS	Probiotic. Tablets. *Bifidobacterium animalis subsp. lactis* BB-12. 5 × 10^9^ CFU. Administered orally via a pacifier.	> 6	The administration of *Bifidobacterium animalis subsp. lactis* BB-12 in childhood may reduce respiratory infections. However, it was not associated with a lower frequency of AOM.
Stecksén-Blicks C et al., 2009 [46]	I:133 C:115	NS	3.5	Probiotic. Milk. *L. rhamnosus* LB21. 1 × 10^7^ CFU in 150 mL of milk. Oral route.	21	Probiotic supplementation did not significantly reduce the days of AOM.
Rautava S et al., 2008 [47]	I:32 C:40	49	<1 year	Probiotic. Infant formula. *L. rhamnosus* GG and *B. lactis* Bb-12. 1 × 10^10^ CFU. Oral route.	10	The results suggest that the probiotics *L. rhamnosus* GG and *B. lactis* Bb-12 are a safe alternative to reduce the risk of early acute otitis media and antibiotic use. In addition, they may decrease the risk of recurrent respiratory infections during the first year of life.
Hatakka K et al., 2007 [48]	I:155 C:154	58	2.4	Probiotic. Capsule. *L. rhamnosus* GG ATCC 53103, *L. rhamnosus* LC 705, *B. breve* 99; *P. freudenreichii* ssp. shermanii JS. 8–9 **×** 10^9^ CFU. Oral route.	6	The probiotics did not prevent otitis or the presence of pathogens in the nasopharynx, although a trend toward a reduction in recurrent respiratory infections was observed, which should be confirmed in future studies.
Arslanoglu S et al., 2007 [49]	I:102 C:104	NS	NS	Prebiotic. Infant formula. Galactooligosaccharides/fructooligosaccharides. 8 g/L. Oral route.	6	The administration of a prebiotic oligosaccharide mixture in early life appears to modulate immunity in the correct direction. However, it was not associated with a lower frequency of acute otitis media episodes.
Hatakka K et al., 2001 [50]	I:252 C:261	51	4.5	Probiotic. Milk. *L. rhamnosus* GG. 5–10 × 10^5^ in 200 mL of milk. Oral route.	7	*Lactobacillus* GG may reduce respiratory infections and their severity among children in daycare. The effects of the probiotic *Lactobacillus* GG were modest. No lower frequency of AOM was observed in the intervention group.

Abbreviations: CFU = colony-forming units; NS = not specified.

**Table 4:** Synthesis of studies evaluating adverse events.

Author, Year	Number of Patients Treated with the Product	Number of Adverse Events (Intervention)	Number of Adverse Events (Control)	Reported Adverse Events	Conclusion on Product Safety
Corsello G et al., 2017 [36]	66	0	0	None	No adverse events related to the consumption of the active products or placebo were recorded.
Di Pierro F et al., 2016 [38]	111	0	0	None	No side effects associated with the treatment were evidenced.
Taipale T et al., 2016 [39]	55	2	1	Gastrointestinal discomfort	No serious adverse events related to the probiotic were reported during the follow-up period.
Hojsak I et al., 2016 [37]	104	0	0	None	No adverse effects were found in the studied population.
Nocerino R et al., 2015 [41]	264	0	0	None	No adverse effects associated with the use of the active products or placebo were recorded throughout the study.
Marchisio P et al., 2015 [40]	50	35	13	Sneezing, itching, cough, nasal congestion, epistaxis, and rhinorrhea	The percentage of children who experienced at least one adverse event was 42% in the intervention group versus 15% in the control group. No systemic or severe adverse events were reported.
Cohen R et al., 2013 [43]	112	4	1	Lack of appetite for milk, regurgitation, dry skin, diarrhea, constipation, and abdominal pain	Most adverse events reported in the participants were not attributed to the treatment. No severe adverse events were reported.
Taipale T et al., 2010 [45]	55	2	1	Gastrointestinal discomfort and dermatological reactions	No serious adverse effects were identified during the probiotic supplementation period.
Stecksén-Blicks C et al., 2009 [46]	133	0	0	None	None of the participants in the study groups reported any intervention-related side effects.
Rautava S et al., 2008 [47]	38	1	3	Vomiting and flatulence	No serious adverse events related to probiotic supplementation were reported during the study.
Hatakka K et al., 2001 [50]	282	0	0	None	No adverse effects associated with the administration of the probiotic product were found in the participants.

**Table 5:** Quality assessment of the evidence based on the Jadad Scale.

Author	The Study Is Randomized	The Intervention Is Double-Blind	Study Withdrawals Are Accounted for and Described	The Randomization Procedure Is Adequately Performed	Selection Criteria	Score
Sarlin S et al., 2023 [34]	1	1	0	1	1	4
Marchisio P et al., 2015 [40]	1	1	1	1	1	5
Cohen R et al., 2013 [43]	1	1	1	1	1	5
Hatakka K et al., 2007 [48]	1	1	0	1	1	4
Rautava S et al., 2008 [47]	1	1	0	1	1	4
Taipale T et al., 2010 [45]	1	1	0	1	1	4
Arslanoglu S et al., 2007 [49]	1	1	0	1	1	4
Garaiova I et al., 2023 [35]	1	1	1	1	1	5
Di Pierro F et al., 2016 [38]	1	0	1	0	1	3
Hatakka K et al. 2001 [50]	1	1	1	1	1	5
Stecksén-Blicks C et al., 2009 [46]	1	1	1	0	1	4
Corsello G et al., 2017 [36]	1	1	1	1	1	5
Hojsak I et al., 2009 [44]	1	1	0	1	1	4
Hojsak I et al., 2016 [37]	1	1	0	1	1	4
Karpova L et al., 2015 [42]	1	0	1	0	1	3
Nocerino R et al., 2015 [41]	1	1	1	1	1	5
Taipale T et al., 2016 [39]	1	1	0	1	1	4

## Data Availability

Data are contained within the article.

## Supplementary Materials

The following supporting information can be downloaded at: https://www.mdpi.com/article/10.3390/children12050591/s1, Figure S1. Sensitivity analysis of the effect of probiotic, prebiotic, or symbiotic therapy on the incidence of acute otitis media, excluding the study by Di Pierro et al. [38]. Figure S2. Sensitivity analysis of the effect of probiotic, prebiotic, or symbiotic therapy on the incidence of acute otitis media, excluding the study by Karpova L et al. [42].
