# Supplementary material for: Probiotic, Prebiotic, and Synbiotic Supplementation for the Prevention and Treatment of Acute Otitis Media: A Systematic Review and Meta-Analysis
Source: Children (Basel). 2025 Apr 30;12(5):591. doi: 10.3390/children12050591 (PMC12109743; doi:10.3390/children12050591)
Supplement: Supplementary file 1 [file children-12-00591-s001.zip › children-3580761-supplementary.pdf]

Supplementary Material, Figure S1. Sensitivity analysis of the effect of probiotic, prebiotic, or symbiotic therapy on the incidence of acute otitis media, excluding the study by Di Pierro et al.

Study or Subgroup	Experimental Events	Experimental Total	Control Events	Control Total	Weight	Risk ratio IV, Random, 95% CI
Arslanoglu et al. 2007	34	413	24	414	6.9%	1.42 [0.86 , 2.35]
Cohen et al. 2013	35	50	40	47	11.3%	0.82 [0.66 , 1.02]
Corsello et al. 2017	80	112	80	112	12.0%	1.00 [0.85 , 1.18]
Garaiova et al.2023	112	155	100	154	12.2%	1.11 [0.96 , 1.29]
Hatakka et al. 2007	7	32	20	40	4.6%	0.44 [0.21 , 0.90]
Hatakka et al.2001	4	102	6	104	2.0%	0.68 [0.20 , 2.34]
Hojsak et al 2016	4	103	5	101	1.9%	0.78 [0.22 , 2.84]
Hojsak et al. 2010	49	111	89	111	11.1%	0.55 [0.44 , 0.69]
Karpova et al.2015	79	252	101	261	11.0%	0.81 [0.64 , 1.03]
Marchisio et al. 2015	8	66	13	60	3.9%	0.56 [0.25 , 1.26]
Nocerino et al.2017	8	139	13	142	3.7%	0.63 [0.27 , 1.47]
Rautava et al. 2008	15	104	18	106	5.5%	0.85 [0.45 , 1.59]
Sarlin et al. 2023	2	113	5	106	1.3%	0.38 [0.07 , 1.89]
Taipale et al 2010	8	264	18	127	4.0%	0.21 [0.10 , 0.48]
Taipale et al.2016	19	31	21	33	8.7%	0.96 [0.66 , 1.41]
**Total (Wald[a])**		**2047**		**1918**	**100.0%**	**0.78 [0.64 , 0.94]**
Total events:	464		553			

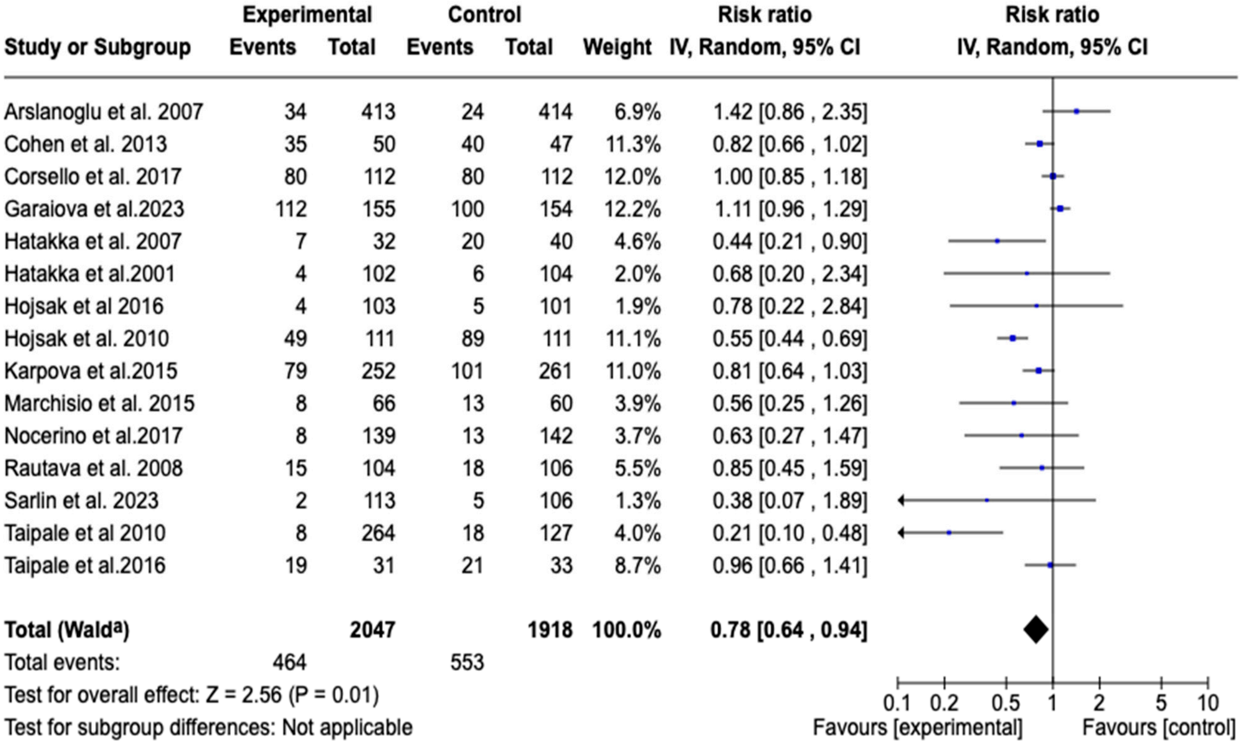

Test for overall effect: Z = 2.56 (P = 0.01)
Test for subgroup differences: Not applicable
Heterogeneity: Tau² (DL[b]) = 0.07; Chi² = 50.66, df = 14 (P < 0.00001); I² = 72%

Footnotes

[a]CI calculated by Wald-type method.
[b]Tau² calculated by DerSimonian and Laird method.

Supplementary Material, Figure S2. Sensitivity analysis of the effect of probiotic, prebiotic, or symbiotic therapy on the incidence of acute otitis media, excluding the study by Karpova L et al.

Study or Subgroup	Experimental Events	Experimental Total	Control Events	Control Total	Weight	Risk ratio IV, Random, 95% CI
Arslanoglu et al. 2007	34	413	24	414	7.6%	1.42 [0.86 , 2.35]
Cohen et al. 2013	35	50	40	47	11.8%	0.82 [0.66 , 1.02]
Corsello et al. 2017	80	112	80	112	12.5%	1.00 [0.85 , 1.18]
Di Pierro et al. 2016	9	34	6	35	3.8%	1.54 [0.62 , 3.87]
Garaiova et al.2023	112	155	100	154	12.6%	1.11 [0.96 , 1.29]
Hatakka et al. 2007	7	32	20	40	5.2%	0.44 [0.21 , 0.90]
Hatakka et al.2001	4	102	6	104	2.4%	0.68 [0.20 , 2.34]
Hojsak et al 2016	4	103	5	101	2.2%	0.78 [0.22 , 2.84]
Hojsak et al. 2010	49	111	89	111	11.6%	0.55 [0.44 , 0.69]
Marchisio et al. 2015	8	66	13	60	4.5%	0.56 [0.25 , 1.26]
Nocerino et al.2017	8	139	13	142	4.2%	0.63 [0.27 , 1.47]
Rautava et al. 2008	15	104	18	106	6.1%	0.85 [0.45 , 1.59]
Sarlin et al. 2023	2	113	5	106	1.5%	0.38 [0.07 , 1.89]
Taipale et al 2010	8	264	18	127	4.5%	0.21 [0.10 , 0.48]
Taipale et al.2016	19	31	21	33	9.4%	0.96 [0.66 , 1.41]
**Total (Wald[a])**		**1829**		**1692**	**100.0%**	**0.79 [0.64 , 0.97]**
Total events:	394		458			

Test for overall effect: Z = 2.21 (P = 0.03)
Test for subgroup differences: Not applicable
Heterogeneity: Tau² (DL[b]) = 0.09; Chi² = 51.49, df = 14 (P < 0.00001); I² = 73%

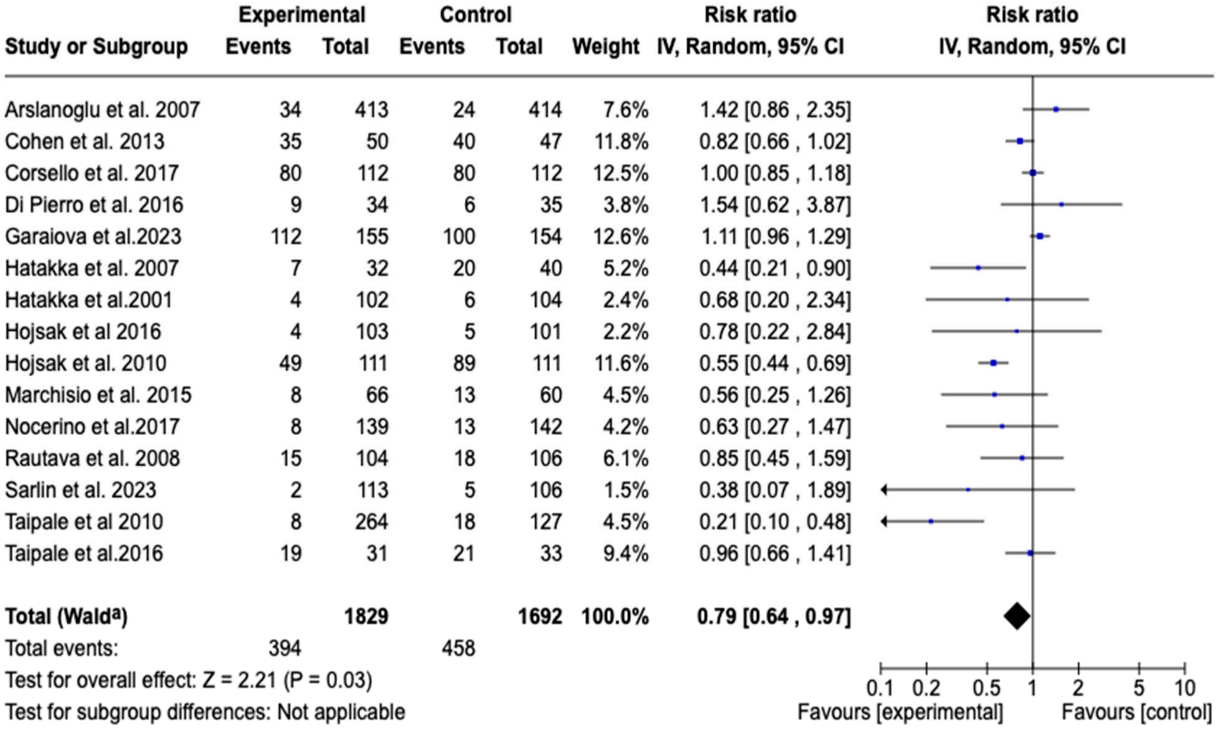

Footnotes
[a]CI calculated by Wald-type method.
[b]Tau² calculated by DerSimonian and Laird method.
